# A case report of a septic hip secondary to a psoas abscess

**DOI:** 10.1186/1749-799X-5-70

**Published:** 2010-09-16

**Authors:** Benan M Dala-Ali, Mary-Anne Lloyd, Satish B Janipireddy, Henry D Atkinson

**Affiliations:** 1 North Middlesex University Hospital, Sterling Way, London- UK; 2 Watford General Hospital, Vicarage Road, Watford, Hertfordshire - UK

## Abstract

Psoas abscess was first described by Mynter in 1881. Though rare, its prevalence is increasing with advances in radiology and an increasing ability to accurately diagnose the condition. The symptoms of a psoas abscess can be insidious and nonspecific, and patients often present with a limp, fever, weight loss, and flank or abdominal pain.

A psoas abscess can be classified as either primary or secondary depending on the presence or absence of an underlying disease. Primary psoas abscess has become more prevalent in the developed world, especially in immuno-compromised patients.

We present the case of a 48 year old man who presented with fever, left hip pain and difficulty weight-bearing. He had a past medical history of chronic renal failure secondary to hypertension. Following laboratory, radiological and microbiological analyses the patient was diagnosed as having a Staphylococcus Aureus hip sepsis secondary to a psoas abscess.

Psoas abscess should be included as a differential diagnosis in all patients presenting with hip pain and constitutional symptoms. The case is discussed with reference to the literature.

## Introduction

A psoas abscess is a collection of pus within the psoas muscle compartment [1]. It is rare in the developed world, but its detection is increasing mainly due to advancements in radiology. These abscesses can be classified as primary (the infection originating in the psoas space) or secondary (as a result of direct extension from the adjacent organs) [2].

We describe the case of a primary psoas abscess in a patient with chronic renal failure in whom the infection spread to the hip joint, leading to a septic arthritis.

## Case Report

A 48-year-old man presented with a 4-day history of right groin pain, fever and rigors, general malaise and anorexia. He had a past medical history of chronic renal failure secondary to hypertension, however was not receiving any renal replacement therapy.

On examination he was mildy pyrexial and haemodynamically stable. His ability to weightbear was impeded by groin pain and he lay in a supine position with the right hip flexed for comfort. There was painful restriction in hip range of motion with discomfort in external/internal rotation and full extension beyond 30 degrees of flexion. There was localised tenderness over the right hip capsule. Abdominal, spinal and other locomotor examination was normal.

**Figure 1:**
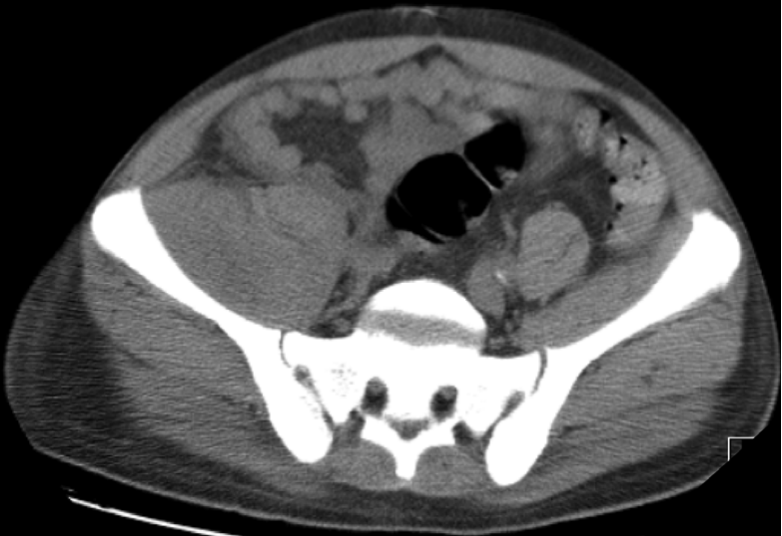
**A CT scan showing hypertrophy of the psoas muscle on the right side**.

Laboratory tests revealed a raised white cell count of 15.7 × 10^9^/l (normal: 4.0-11.0 × 10^9^/l) and a C-reactive protein of 586 mg/l (normal <5 mg/l). Creatinine was significantly raised at 1133 mmol/l (42-102 mmol/l). Urinalysis and urine culture were negative and a chest radiograph was clear. CT scan identified a large right psoas abscess (Figure 1).

The patient was haemofiltered and underwent CT-guided percutaneous abscess drainage. He was initially commenced on intravenous Tazocin^® ^4.5 g TDS (Piperacillin and Tazobactam), while awaiting microbiological analyses. Blood cultures and the abscess aspirate subsequently both grew *Staphyloccous aureus *sensitive to Flucloxacillin, and the patient was converted to intravenous Flucloxacillin and oral Fusidic acid.

**Figure 2:**
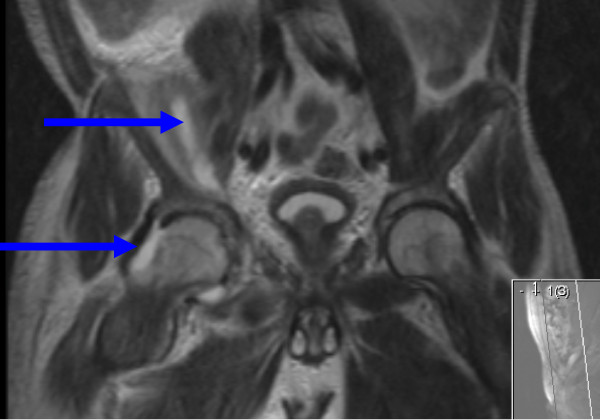
**A T2 weighted MRI image of the pelvis in the coronal plane**. This image demonstrates an area of high signal around the right iliacus and psoas muscles. It also shows evidence of fluid in the hip joint.

The patient responded well to antibiotics and after 5 days was comfortably independently mobilising. Inflammatory markers and renal function normalised by 10 days and a follow-up MRI scan (at 10 days) showed a significantly reduced and resolving collection. However, the scan also revealed that the collection had also tracked into the right hip joint (Figure 2). In view of the resolving clinical picture, an expectant treatment pathway was taken. The patient was discharged home clinically well after 2 weeks and remains well at 6 month follow-up.

## Discussion

The psoas abscess was first described in 1881, and referred to as psoitis [3]. Its estimated worldwide incidence was 3.9 cases per year in 1985 [4], and 12 cases per year in 1992, a rise believed to be attributed more to improvements in radiological imaging rather than a true increase in the incidence of the condition [5].

An understanding of the anatomy of the psoas muscle helps to explain the clinical manifestations of the psoas abscess. The muscle arises from the transverse processes and lateral aspects of the vertebral bodies between T12 and L5. It then travels downwards across the pelvic brim to insert on the lesser trochanter of the femur. The muscle lies within the retroperitoneal space close to the sigmoid colon, jejunum, appendix, ureters, iliac lymph nodes, abdominal aorta and the hip capsule. Thus infections can spread between these structures and the psoas muscle. A bursa is also present between the psoas muscle tendon insertion at the lesser trochanter and the hip capsule. This bursa is a potential route by which an infection from a psoas abscess can gain access to the hip joint. Infection can also track to the hip capsule directly along the iliopsoas muscle between the iliofemoral and iliopubic ligaments [6]. The psoas has a rich blood supply that is believed to predispose it to haematogenous spread from sites of occult infection [2]. It is innervated by branches of the L2, L3 and L4 nerves; thus pain often radiates to the anterior thigh.

Primary psoas abscesses are more commonly found in the developing world. A study found that 99.5% of psoas abscesses in Africa and Asia were primary [7]. However there has been an increase in recent years in the developed world, especially in immunocompromised patients. This includes patients infected with the human immunodeficiency virus (HIV), intravenous drug users and patients with chronic illnesses such as diabetes or renal failure [8,9]. Secondary psoas abscesses occur when there is a direct extension from an adjacent retroperitoneal or intra-abdominal infection. The most common cause of secondary abscesses in the literature is spread from the gastro-intestinal tract, such as in Crohn's disease, appendicitis, diverticulitis and colon cancer. Other causes of secondary abscesses include vertebral osteomyelitis and urinary tract infections [7].

The symptoms of a psoas abscess can be insidious and non-specific. Patients often present with fever, weight loss, limp, flank or abdominal pain. The key to the diagnosis lies in the physical examination. The patient is classically more comfortable lying supine with the leg slightly flexed and externally rotated. The pain is often exacerbated by flexing the hip against resistance.

Laboratory tests may reveal a raised white cell count and erythrocyte sedimentation rate. The patient may also be anaemic. Radiological images should be used to make the diagnosis. A CT scan of the abdomen and pelvis is the image of choice and is diagnostic in 80-100% of cases [10-12], compared to 60% with ultrasonography [2]. The CT scan also helps to identify the aetiology and can guide the percutaneous drainage/aspiration of the abscess. A scan of a psoas abscess would reveal an enlarged psoas muscle belly compared to the unaffected other side. They may also show focal areas of low density and/or gas within the affected region [13].

The bacteriology is different in a primary and secondary abscess. *Staphylococcus aureus *is the pathogen in 88% of primary psoas abscesses [7]; other pathogens include *streptococci*, *Escherichia coli *[7], *Pseudomonas aeruginosa *[8] and *Proteus mirabilis *[14]. Cultures are often mixed in secondary abscesses, with *E. coli *and *bacteriodes spp *occurring most commonly. Other pathogens include *Staphylococcus *and *Streptococcus spp.*[7]. *Mycobacteria tuberculosis *is also a frequent causative organism in areas where it is endemic, however is rare in the developed world.

The management is through antibiotic treatment accompanied by abscess drainage. Broad spectrum antibiotics should initially be commenced until microbiology cultures of the abscess fluid hone in on the specific anti-microbial sensitivities. Initial coverage should include staphylococcal and enteric organisms for which agents such as clindamycin, antistaphylococcal penicillin, and an aminoglycoside may be used [12]. Drainage of the abscess can be performed surgically or percutaneously. CT-guided percutaneous drainage is the method of choice [11] and should be utilised where possible. However surgical drainage may be advantageous if the abscess is secondary to gastro-intestinal disease, as it also provides an opportunity to resect the diseased bowel [11]. The prognosis is generally good. Primary psoas abscess has a better prognosis than secondary. The most common cause of death is due to delayed or inadequate treatment, hence the emphasis on an early diagnosis. Mortality from an undrained abscess is close to 100%, with septicaemia being the most common cause of death [4].

Our patient had a rare presentation of a psoas abscess where the infection tracked into the hip joint. There have been only a very small number of similar documented cases [6,15-17], and some were associated with complications such as pathological hip fracture [17]. It is also possible for the infection to spread in the opposite direction, namely the primary infection being within the hip, which then extends through the capsule into the psoas to form an abscess. Half of patients with a septic joint go on to develop permanent joint damage and there is an associated mortality rate of 10-16% [18-20]. Our patient did not suffer any hip sequelae and remains clinical well at 6 months, possibly a reflection of the early diagnosis and commencement of definitive treatment.

## Conclusion

Psoas abscesses are becoming more frequently diagnosed in the Western world. It is essential that all orthopaedic surgeons are aware of the condition. The symptoms are often subtle and can easily be missed. A good clinical examination is the key, followed by a CT scan to definitively identify the abscess and its possible aetiology. Treatment is with both antibiotics and drainage and is usually curative.

## Consent

Written informed consent was obtained from the patient for publication of this case report and any accompanying images.

## Competing interests

The authors received no financial or other type of support to carry out this study; there is no conflict of interests.

This is an original article and has not been published in any other journal.

## Authors' contributions

BD, SJ and HA managed the patient. BD and ML wrote the manuscript. SJ and HA assisted with the literature review and manuscript preparation. All authors have read and approved the final manuscript.
